# Study of X-ray topography using the super-Borrmann effect

**DOI:** 10.1107/S1600577522007779

**Published:** 2022-08-17

**Authors:** J. Matsui, K. Takatsu, Y. Tsusaka

**Affiliations:** a Synchrotron Radiation Research Center, Hyogo Science and Technology Association, 1-490-2 Kouto, Shingu, Tatsuno, Hyogo 679-5165, Japan; b Graduate School of Science, University of Hyogo, 3-2-1 Kouto, Kamigori, Hyogo 678-1297, Japan; University of Tokyo, Japan

**Keywords:** topography, super-Borrmann effect

## Abstract

X-ray topography exerting the super-Borrmann effect has been performed using synchrotron radiation to display dislocation images with a high-speed and high-resolution CMOS camera. Forward-transmitted X-rays are positively employed instead of reflected X-rays to reveal dislocations in relatively thick crystals by simultaneously exciting a pair of adjacent {111} planes owing to the super-Borrmann effect. Before the experiment, minimum values of the attenuation coefficients *A*_min_^*P*^ for σ and π polarizations of the incident X-rays in the three-beam case are calculated. Results demonstrate that *A*_min_^*P*^ for both polarizations are almost 20 times larger than those in the two-beam (usual Borrmann effect) case. The transmitted X-rays can be used to confirm the efficacy of taking topographs under the super-Borrmann conditions, as well as under multiple-diffraction conditions. Furthermore, super-Borrmann topographs can be considered for relatively thick crystals, where a conventional Lang X-ray topography technique is difficult to apply.

## Abstract 2

X-ray topography exerting the super-Borrnann effect is realized using synchrotron radiation and a high-resolution CMOS camera.

## 1. Introduction

The effect of anomalous transmission can be enhanced (Borrmann & Hartwig, 1965 ▸) if the Bragg condition is satisfied for the 111 and 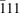 reflections simultaneously in the wide-angle diagram of perfect germanium (Ge) crystals with thickness *t* = 0.8 mm and 1.2 mm with Cu *K*α radiation. Enhanced intensity spots for the 111 and 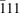 reflections appear at the Kossel line intersection of the *T*_111_ and 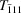 traces of the reflected beams, respectively. Furthermore, enhanced intensity spots for the 111 and 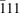 reflections appear on the *R*_111_ and 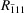 traces of the transmitted (refracted in the strict sense) beams, respectively, and are symmetrical to the intersection point of the *T*_111_ and 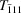 traces with respect to the respective reflecting planes. Calculation results and interpretation for the decrease in the absorption coefficient was provided (Hildebrandt, 1966 ▸, 1967 ▸) for the enhanced spots in the 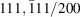 three-beam case (/*hkl* means χ_*g*–*h*_ when *g* = 111 and *h* = 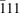 ). Later, the theoretical understanding based on detailed calculation was advanced (Feldman & Post, 1972 ▸), and was confirmed experimentally (Uebach & Hilde­brandt, 1969 ▸; Hildebrandt, 1978 ▸).

Other combinations of simultaneous reflections for three-beam cases such as 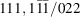 (Umeno & Hildebrandt, 1975 ▸) and 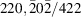 (Umeno, 1972 ▸) were investigated. The Borrmann effect for four-beam and six-beam cases involving 220 reflections was found to be also enhanced (Joko & Fukuhara, 1967 ▸). Theoretical explanations were also discussed by Afanasev & Kohn (1975 ▸, 1976 ▸, 1977 ▸). This enhanced Borrmann effect is called the ‘super-Borrmann’ effect (Lang, 1998 ▸).

In terms of the application of the super-Borrmann effect to X-ray topography to image lattice defects in crystals, such as dislocations, as well as conventional X-ray topography, few reports have been published probably owing to a too low X-ray source intensity and large X-ray beam divergence to develop clear defect images for a wide visual field. However, since the availability of synchrotron radiation, X-ray topography can be applied for imaging lattice defects in crystals by choosing the correct X-ray wavelength. In addition, it was previously reported that topographs combined with a high-speed and high-resolution CMOS camera taken by employing forward-transmitted X-rays under multiple diffraction conditions (bright-field X-ray topographs) can reveal dislocations without noticeable image deformations (Tsusaka *et al.*, 2016 ▸, 2019 ▸).

Furthermore, as a major advantage, it is expected that the forward-transmitted X-rays riding on the super-Borrmann effect also reveal dislocations existing in relatively thick crystals by simultaneously exciting a pair of adjacent {111} planes such as (111) and 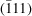 where conventional Lang X-ray topography is difficult to apply. Therefore, this study deals with X-ray topography performed under three-beam multiple-diffraction conditions for thick Ge crystals using synchrotron radiation.

## 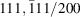 three-beam case

2.

**Figure 1:**
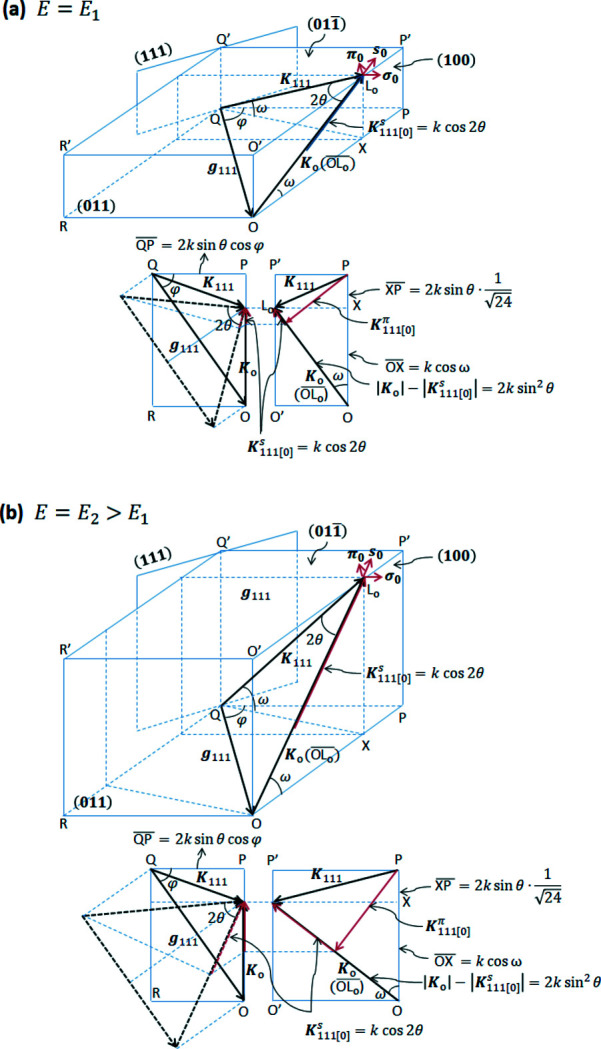
Schematic of 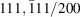 three-beam multiple diffraction in reciprocal space of Ge, where only the 111 reflection is represented, considering the 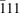 beam travels in a symmetrical direction with the plane of symmetry (100) at incident X-ray energies of *E* = *E*_1_ in (*a*) and *E* = *E*_2_ (>*E*_1_) in (*b*) with rectangles *OPQR* and *PP′O′O* representing projections on the 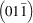 and (100) planes, respectively. 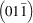 : entrance surface; **K**_o_: incident X-ray wavevector; **K**_111_: 111-reflected X-ray wavevector; **g**_111_: diffraction vector of the 111 reflection.

Fig. 1 ▸ shows an example of 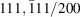 three-beam multiple diffraction in reciprocal space of a perfect Ge crystal. Note that the 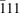 beam is not drawn here considering that it passes symmetrical to the 111 beam with respective to the (100) plane of symmetry in order to simplify calculation of the absorption decreases controlling the super-Borrmann effect. Figs. 1 ▸(*a*) and 1(*b*) demonstrate two cases of different energy of the incident X-rays for *E* = *E*_1_ and *E* = *E*_2_, respectively, which is higher than *E*_1_. The black dashed triangle in Fig. 1 ▸ comprises the original **K**_o_, **K**_111_ and **g**_111_, where **K**_o_ is the incident X-ray wavevector, **K**_111_ is the 111-reflected X-ray wavevector and **g**_111_ = **K**_111_ − **K**_o_ is the diffraction vector of the 111 reflection lying on the same plane. Rectangles *OPQR* and *PP*′*O*′*O* represent projections on 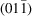 and (100), respectively. The lengths of sides 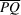 , 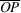 and 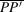 are given as follows,

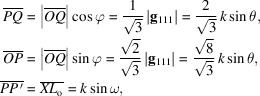

where ω is an elevation angle of **K**_o_ (or **K**_111_) from the rectangle *OPQR* parallel to the 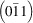 entrance surface. It is clear that both 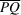 and 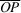 are independent of *E*; however, 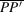 becomes larger when *E* increases, as observed in Figs. 1 ▸(*a*) and 1(*b*). Then, we put a unit vector of **K**_o_ as **s**_o_ and unit vectors of the polarization components of **K**_o_ as **σ**_o_ and **π**_o_, for horizontal and vertical polarizations, respectively. **σ**_o_ lies in the 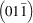 base plane and is perpendicular to **s**_o_. Therefore, **π**_o_ is also perpendicular to **s**_o_ and **σ**_o_.

Next, we put 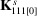 , 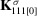 and 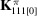 in Fig. 1 ▸ as components of **K**_111_ in the **s**_o_, **σ**_o_ and **π**_o_ directions, respectively. The magnitudes of these vectors were calculated as 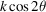 , 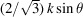 and 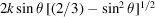 , respectively. The lengths of sides 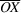 and 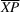 were found to have the following values,

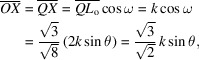




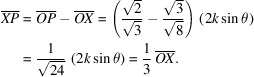

Then, we obtained 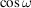 = 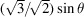 and 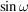 = 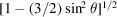 as a result for the plane parallel to the 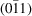 entrance surface.

Since 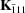 is symmetrical with **K**_111_, 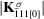 = 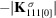 ; however, the **s**_o_, **π**_o_ components of 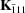 and **K**_111_ are identical. Consequently, the refracted beam **K**_o_ and two reflected beams **K**_111_ and 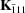 are summarized as follows,

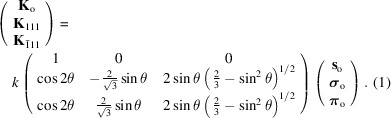




## 3. Absorption coefficients in the three-beam diffraction cases

Since the calculation process for the absorption coefficient in the three-beam case under Cu *K*α_1_ radiation has already been provided by Authier (2001 ▸), explanation of the calculation will be kept to a minimum. Based on the fundamental equations of X-ray dynamical theory, the projections of the electric displacements **D** in the three-beam case to the plane normal to **K**_o_ can be written for the three beams as follows,

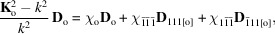




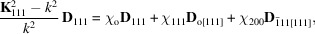




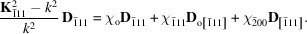

It is possible to rewrite the above equations by using excitation errors 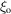 , 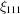 , 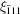 ,

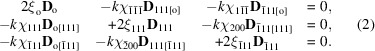

Since the 200 reflection and 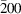 reflectin are forbidden ( 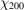 = 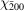 = 0), the above equations are expressed as follows,

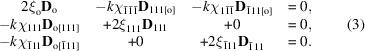

The two relations are obtained from the second and third lines of equation (3) shown above,

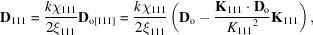




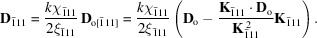

From **D**_o[111]_ and 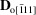 one can obtain **D**_111[o]_ and 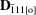 using a vector formula **A** × (**B** × **C**) = (**A** · **C**)**B** − (**A** · **B**)**C** in a similar way to that described by Authier (2001 ▸). By substituting **D**_111[o]_ and 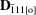 thus obtained in the first line of equation (3), we obtain a relation involving only **D**_o_. If we decompose **D**_o_ into two components, 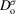 , parallel to the plane of symmetry, and 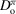 , perpendicular to the plane of symmetry,

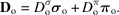

The first line of equation (2), which is

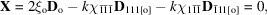

can be replaced using two scalar values 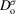 and 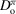 , given as follows,

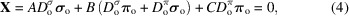

where *A*, *B* and *C* are the coefficients of 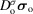 , 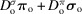 and 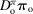 , respectively,

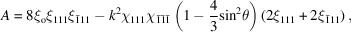




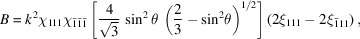




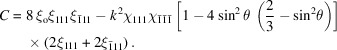

Considering **X** can be separated into **σ**_o_ and **π**_o_,

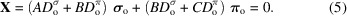

Therefore, we can derive the determinant as follows,

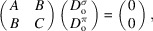

and hence, inevitably,

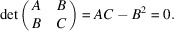

Note that the components of **K**_o_, **K**_111_ and 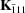 are given by equation (1) and 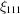 = 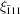 . Therefore, we understand that *B* vanishes, and hence *AC* also vanishes.

From the above considerations, *A* (coefficient of 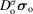 ) and *C* (coefficient of 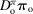 ) should be null independently for **σ**_o_ and **π**_o_ polarizations, respectively. As a result, we obtain

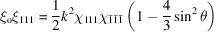

for **σ**_o_ polarization and

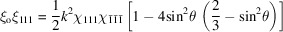

for **π**_o_ polarization.

Considering χ_o_ is minimum when |ξ_o_| = |ξ_111_|, the subsequent values for ξ_o_ can be given as

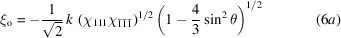

for **σ**_o_ polarization and

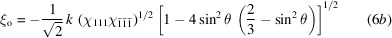

for **π**_o_ polarization.

In the two-beam case,

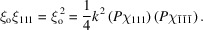

When |ξ_o_| = |ξ_111_|, satisfying the Bragg condition exactly,

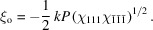

For a cubic crystal such as Ge, the structure factor *F*_111_ and Fourier component of the dielectric susceptibility χ_111_ for the 111 reflection are given as follows,

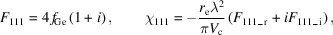

where *f*_Ge_ is the atomic scattering factor of Ge, *r*_e_ is the classical electron radius, λ is the wavelength of the X-rays, and *V*_c_ is the volume of a unit cell of Ge. *F*_111_r_ and *F*_111_i_ are the real and imaginary parts of the complex number *F*_111_, respectively. The magnitudes of *F*_111_ and χ_111_ can be derived from the corresponding atomic scattering factors as follows,

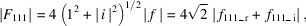




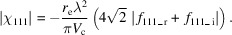

Then,

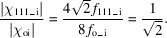

The minimum value of the absorption coefficient in the *g*, 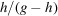 = 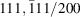 three-beam case is given as follows,

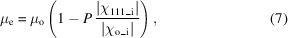

where μ_o_ is the normal absorption coefficient and χ_111_i_ and χ_o_i_ are imaginary parts of χ_111_ and χ_o_, respectively.

**Figure 2:**
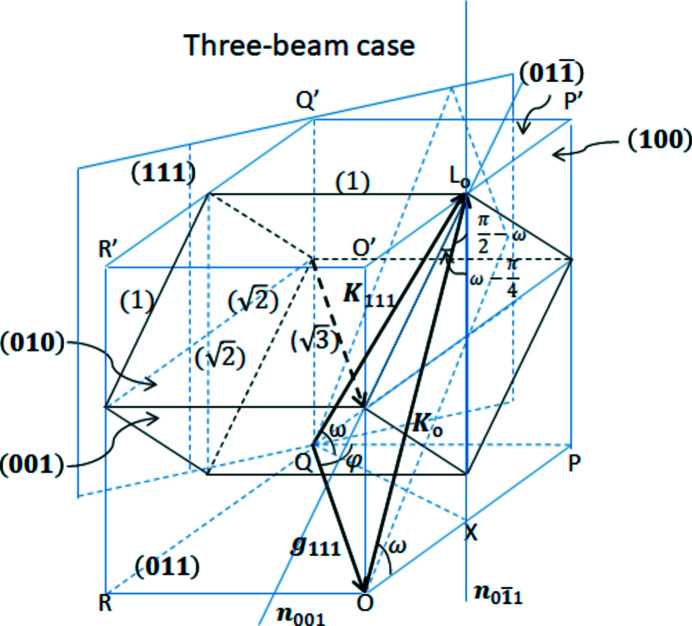
Schematic of the relation between **K**_o_, the wavevector of the incident X-rays, and **n**_001_, the (001) surface normal, or 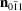 , the 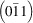 surface normal. Angles 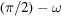 and 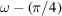 correspond to direction cosines γ_o_ of **K**_o_ to **n**_001_ and 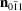 , respectively.

The minimum attenuation coefficient is 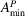 = 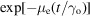 , where *t* is the slab thickness and γ_o_ = **n**_*hkl*_ · **s**_o_ is a direction cosine of the incident X-ray wavevector **K**_o_ (its unit vector is **s**_o_) to **n**_*hkl*_, the normal to the X-ray entrance surface. In the 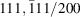 present three-beam case, it is found from Fig. 2 ▸ that the direction cosine γ_o_ is expressed as

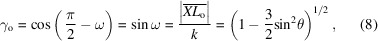

for **K**_o_ to 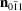 .

However, the X-ray energy in the present experimental case using synchrotron radiation was *E* = 10 keV and the Ge slab thickness was *t* = 0.05 cm with the (100) entrance surface. Because the lattice parameter of Ge is *a* = 0.56754 nm and the Bragg angle of the 111 reflection becomes 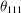 = 7.2458° leading to 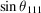 = 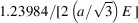 = 0.12613, we can derive γ_o_ for **n**_*hkl*_ = **n**_001_ from Fig. 2 ▸ as follows,

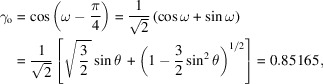

which corresponds to 31.608° as an angle between **n**_001_ and **s**_o_. In this case, the polarization factors *P* for the **σ**_o_ and **π**_o_ components are introduced from equation (6) as

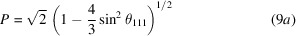

for **σ**_o_ polarization and

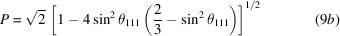

for **π**_o_ polarization

This makes it possible to calculate the effective absorption coefficient μ_e_ and the minimum attenuation coefficient 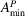 in the 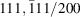 three-beam case for the Ge slab having the (001) entrance surface with thickness of 0.05 cm, as demonstrated in Table 1 ▸, by retrieving the data on the attenuation length from CXRO (https://henke.lbl.gov/optical_constants/atten2.html). Furthermore, it was observed that 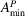 for both polarizations in the three-beam case are approximately 20 times the values in the two-beam case, due to which the phenomenon is called the super-Borrmann effect.

## 4. Topography experiment using the super-Borrmann effect for Ge crystals

**Figure 3:**
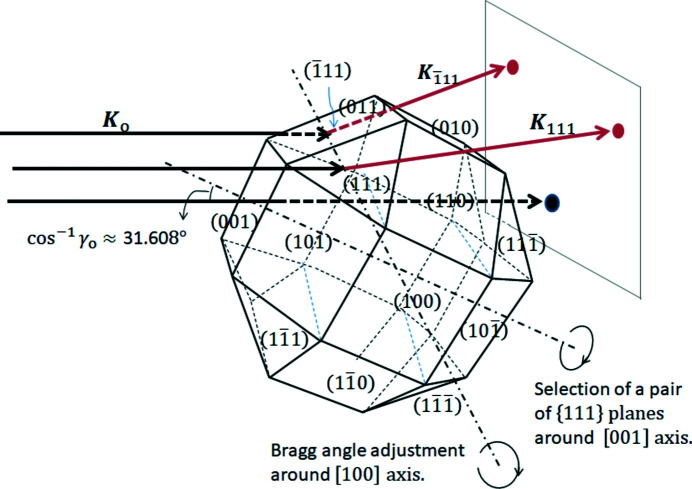
Incident X-rays **K**_o_ and reflected X-rays **K**_111_ and 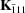 simultaneously satisfying the Bragg conditions for the 111 and 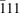 reflections, respectively, where sample rotations around [001] and [100] are performed, respectively, to select a pair of {111} planes and for Bragg condition adjustment to satisfy the individual three-beam diffraction condition.

To take X-ray topographs with minimized image deformation, we employed forward-transmitted (but refracted) X-rays that satisfied the Bragg conditions for the two {111} adjacent planes lying symmetrically with respect to the {100} plane of symmetry, as demonstrated in Fig. 3 ▸. An X-ray diffraction goniometer with an X-ray source of approximately 1.2 mm × 1.2 mm was used with 10 keV X-rays from the synchrotron radiation through a silicon double-crystal monochromator at the BL24XU8 beamline of SPring-8 (Tsusaka *et al.*, 2001 ▸), similar to previously reported multiple-beam diffraction topography (Tsusaka *et al.*, 2016 ▸, 2019 ▸). In order to avoid the harmonics of the incident synchrotron beam, the usual detuning treatment was carried out before carrying out the topography experiment.

Various interference patterns on diffracted and transmitted images with defect appearance were also studied using a coherent X-ray beam under multiple-diffraction conditions (Okitsu *et al.*, 2003 ▸; Okitsu, 2003 ▸). However, in the present case, topographic images were taken directly by the forward-transmitted X-ray beam instead of the diffracted X-ray beam using an X-ray imaging detector (Hondoh *et al.*, 1989 ▸). The detector comprises a 20 µm-thick Gd_3_Al_2_Ga_3_O_12_ (GAGG) scintillator, relay lens optics and a high-speed CMOS camera (Hamamatsu, C11440-22CU). This detector resolved a 1 µm line-and-space pattern.

A Ge slab of dimensions 10 mm (width) × 14 mm (height) × 0.5 mm (thickness) and the (001) surface was prepared for the super-Borrmann topography experiment. The slab was rotated in the clockwise direction around the [100] axis until bright spots corresponding to the reflections from two adjacent {111} planes, for example (111) and 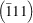 , could be recognized on a fluorescent sheet. It is clear that this multiple (*n*-beam) diffraction from a single crystal is not considered to be so-called *umweganregung* (Reninger, 1937*a* ▸,*b* ▸) but simply simultaneous excitation of the plural diffractions. After confirming the double fluorescent spots by the two 111 reflections on the sheet, the images formed by the forward-transmitted beam were directly captured by the CMOS camera. As demonstrated in Fig. 3 ▸, an adjacent pair of {111} planes was selected by rotating the slab 90° clockwise around the normal to the (001) slab surface.

**Figure 4:**
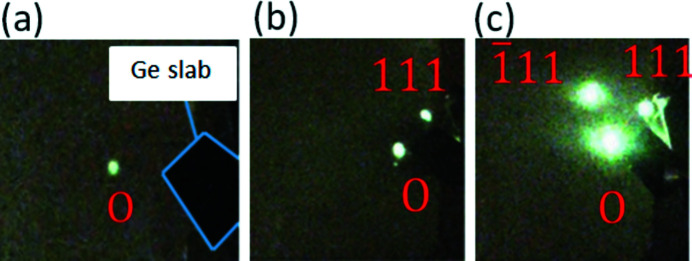
Reflection spots on a fluorescent sheet. (*a*) Directly transmitted X-ray beam denoted as ‘0’, (*b*) direct beam and 111 reflected beam (suggesting the usual Borrmann case) and (*c*) direct beam and 111 and 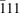 reflected beams (suggesting the super-Borrmann case).

Figs. 4 ▸(*a*)–4(*c*) show a fluorescent spot from (*a*) the directly transmitted X-ray beam denoted as ‘0’, (*b*) the direct beam and the 111 reflected beam, and (*c*) the direct beam, the 111 reflected beam and the 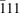 reflected beam. It can be easily noticed that the triple fluorescent spots in Fig. 4 ▸(*c*) are much brighter than those in Fig. 4(*b*), indicating the super-Borrmann effect. The shining light on the right-hand side of Fig. 4 ▸(*c*) is due to a specular reflection by the Ge crystal surface from the 111 reflection spot on the fluorescent sheet. After the triple fluorescent spots were recognized with nearly the same brightness by sample rotation adjustment around [100] and [001], the topographic image formed by the transmitted beam was captured by the CMOS camera. During the usual Borrmann topography adjustment procedure, no clear dislocation images were recognized on the monitor.

**Figure 5:**
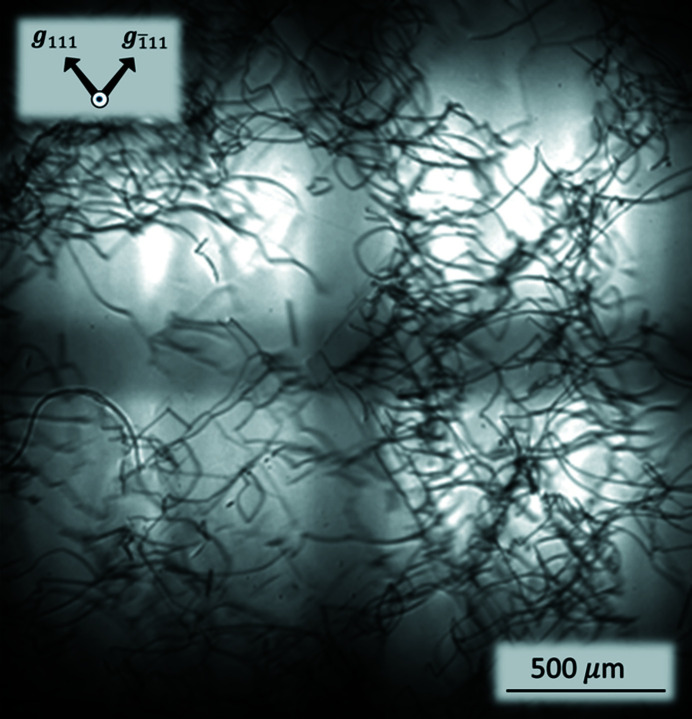
X-ray topograph of a germanium slab taken by simultaneous 111 and 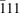 reflections without deformation correction. Four shots of images are pasted together to obtain a wide-area topograph.

Fig. 5 ▸ shows one of the topographs taken under the super-Borrmann conditions shown in Fig. 4 ▸(*c*) using a pair of 111 and 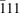 reflections without deformation correction. Considering the X-ray source is approximately 1.2 mm × 1.2 mm in size, four shots of topographic images are pasted together to achieve a wide-area topograph. Regarding the usual Borrmann topography (two-beam case), the images of the dis­locations correspond to local lower transmitted intensities (in the forward-refracted direction) or lower diffraction intensities (along the diffraction direction), since crystal imperfection can destroy the Borrmann effect. This is also true for the current super-Borrmann topography (three-beam case), since double excitation of the 111 and 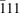 reflections only enhances the Borrmann effect, *i.e.* black contrast on the camera monitor corresponds to the local lower diffraction intensity and white contrast corresponds to the local higher intensity, a phenomenon contradictory to that on negative film.

**Figure 6:**
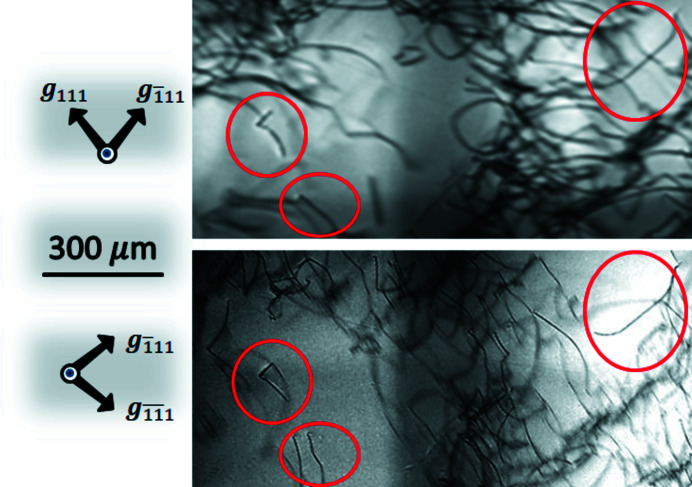
X-ray topographs of a germanium slab taken by (*a*) simultaneous 111 and 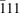 reflections and (*b*) simultaneous 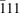 and 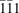 reflections with deformation correction where dislocation configurations circled in red in (*a*) and (*b*) correspond to each other.

There are four combinations of two adjacent 111 reflections, *i.e.* 111 and 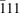 reflections (called A-type), 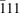 and 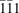 reflections (B-type), 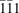 and 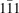 reflections (C-type), and 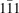 and 111 reflections (D-type). Additionally, there are two combinations of diagonal 111 reflections, *i.e.* 111 and 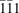 reflections (E-type), and 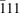 and 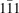 reflections (F-type). Therefore, if one observes dislocation images disappearing only in an A-type topograph, the dislocation should have a Burgers vector of 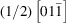 , considering this vector is commonly perpendicular to both [111] and 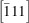 . Similarly, from the invisibility rule **g** · **b** = 0, where **g** is the diffraction vector and **b** the dislocation Burgers vector, B-, C- and D-type topographs do not include any images of the dislocations with Burgers vectors of, respectively, ( 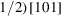 , 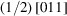 and 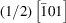 . However, the combination of diagonal 111 reflections (E- and F-type) does not develop into the super-Borrmann effect owing to the existence of χ_220_ instead of χ_200_ in equation (2). According to the partial lack of the super-Borrmann conditions, Burgers vectors of all the dislocations cannot be determined completely by only observing the A-, B-, C- and D-type topographs. Nevertheless, we can conclude that the Burgers vector of the dislocations dis­appearing only on the A-type topograph should belong to 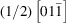 . For example, some parts of A- and B-type topographs are shown in Figs. 6 ▸(*a*) and 6 ▸(*b*), respectively, for the same part of the specimen. The dislocation configurations circled in red can be seen in both topographs.

## 5. Conclusions

In this study, we conducted synchrotron X-ray topography exerting the super-Borrmann effect for imaging dislocations using a CMOS camera. Forward-transmitted X-rays can reveal dislocations in relatively thick crystals by simultaneously exciting a pair of adjacent {111} planes owing to the super-Borrmann effect. Super-Borrmann topographs can be captured for relatively thick crystals, even when a conventional Lang X-ray topography technique is difficult to apply.

Prior to the experiment, the minimum attenuation coefficients 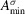 and 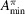 for σ- and π-polarizations, respectively, of the incident X-rays in the three-beam (super-Borrmann) case were calculated. It was found that 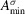 and 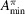 were almost 20 times larger than those in the two-beam (usual Borrmann effect) case.

Although it is possible to determine Burgers vectors for some of the dislocations based on the invisibility criteria, it is difficult to finalize the Burgers vectors of most dislocations considering that the employment of a pair of diagonal {111} planes does not produce the super-Borrmann effect.

In addition to the topographs taken by employing forward-transmitted X-rays under multiple-diffraction conditions (bright-field X-ray topographs), the forward-transmitted X-rays riding on the super-Borrmann effect also reveal dislocations existing in comparatively thick crystals by simultaneously exciting a pair of adjacent {111} planes such as (111) and 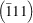 . Therefore, this study deals with X-ray topography using synchrotron radiation performed under three-beam multiple diffraction conditions exerting the super-Borrmann effect for thick Ge crystals. Future research will attempt to experimentally detect dislocation behaviors around the very initial growth stage in the necking parts of dislocation-free silicon crystals.

It was clarified that forward-transmitted X-rays using synchrotron radiation can be used to confirm the efficacy for capturing topographs not only under usual multiple diffraction conditions but also under super-Borrmann conditions.

## Figures and Tables

**Table 1:** μ_o_, μ_e_, 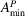 for **σ**_o_ and **π**_o_ polarizations in the 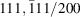 three-beam reflection case for a Ge slab (thickness: 0.05 cm; X-ray entrance surface: (100); X-ray energy: 10 keV)

Polarization	Linear absorption coefficient μ_o_	Polarization component *P*	Effective absorption coefficient μ_e_	Minimum attenuation coefficient 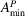 = 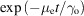
**σ**_o_	192.213	1.380	4.638	0.672
**π**_o_		1.349	8.878	0.594
